# Tumour T_1_ changes *in vivo* are highly predictive of response to chemotherapy and reflect the number of viable tumour cells – a preclinical MR study in mice

**DOI:** 10.1186/1471-2407-14-88

**Published:** 2014-02-14

**Authors:** Claudia Weidensteiner, Peter R Allegrini, Melanie Sticker-Jantscheff, Vincent Romanet, Stephane Ferretti, Paul MJ McSheehy

**Affiliations:** 1 Oncology Research, Novartis Institutes for Biomedical Research, Basel, Switzerland; 2 Global Imaging Group, Novartis Institutes for Biomedical Research, Basel, Switzerland; 3 Department of Radiology Medical Physics, University Medical Center Freiburg, Magnetic Resonance Development and Application Center, Breisacher Str. 60a, 79106 Freiburg, Germany

**Keywords:** Biomarkers, MRI, MRS, T_1_, Animal models, Everolimus, Tumour

## Abstract

**Background:**

Effective chemotherapy rapidly reduces the spin–lattice relaxation of water protons (T_1_) in solid tumours and this change (ΔT_1_) often precedes and strongly correlates with the eventual change in tumour volume (TVol). To understand the biological nature of ΔT_1_, we have performed studies *in vivo* and *ex vivo* with the allosteric mTOR inhibitor, everolimus.

**Methods:**

Mice bearing RIF-1 tumours were studied by magnetic resonance imaging (MRI) to determine TVol and T_1_, and MR spectroscopy (MRS) to determine levels of the proliferation marker choline and levels of lipid apoptosis markers, prior to and 5 days (endpoint) after daily treatment with vehicle or everolimus (10 mg/kg). At the endpoint, tumours were ablated and an entire section analysed for cellular and necrotic quantification and staining for the proliferation antigen Ki67 and cleaved-caspase-3 as a measure of apoptosis. The number of blood-vessels (BV) was evaluated by CD31 staining. Mice bearing B16/BL6 melanoma tumours were studied by MRI to determine T_1_ under similar everolimus treatment. At the endpoint, cell bioluminescence of the tumours was measured *ex vivo*.

**Results:**

Everolimus blocked RIF-1 tumour growth and significantly reduced tumour T_1_ and total choline (Cho) levels, and increased polyunsaturated fatty-acids which are markers of apoptosis. Immunohistochemistry showed that everolimus reduced the %Ki67^+^ cells but did not affect caspase-3 apoptosis, necrosis, BV-number or cell density. The change in T_1_ (ΔT_1_) correlated strongly with the changes in TVol and Cho and %Ki67^+^. In B16/BL6 tumours, everolimus also decreased T_1_ and this correlated with cell bioluminescence; another marker of cell viability. Receiver-operating-characteristic curves (ROC) for everolimus on RIF-1 tumours showed that ΔT_1_ had very high levels of sensitivity and specificity (ROC_AUC_ = 0.84) and this was confirmed for the cytotoxic patupilone in the same tumour model (ROC_AUC_ = 0.97).

**Conclusion:**

These studies suggest that ΔT_1_ is not a measure of cell density but reflects the decreased number of remaining viable and proliferating tumour cells due to perhaps cell and tissue destruction releasing proteins and/or metals that cause T_1_ relaxation. ΔT_1_ is a highly sensitive and specific predictor of response. This MRI method provides the opportunity to stratify a patient population during tumour therapy in the clinic.

## Background

Biomarkers are crucial to the development of new drugs and optimization of the existing options, by facilitating selection of the population to treat, confirming proof-of-concept and acting as early markers of tumour-response. The latter can be provided in the clinic by non-invasive functional imaging, for example positron emission tomography (PET) measurements of 2′-deoxy-2′-[18 F]fluoro-glucose (FDG) and 3′-deoxy-3′-[18 F]fluorothymidine (FLT), dynamic contrast-enhanced magnetic resonance imaging for vascular parameters and diffusion-weighted imaging for apoptosis [1,2]. However, they are not always easy to implement, and furthermore may be inappropriate for the mechanism-of-action (MoA) of a particular drug and cannot always detect true responses to the treatment [3-6]. We have recently described a rapid, robust MRI-method, which detects the response of solid tumours to drugs with different MoA in several different experimental models [7]. The method quantifies the spin–lattice relaxation of protons (T_1_) in tumours both rapidly and accurately using an IR-TrueFISP method. Across several models, the fractional change in tumour T_1_ (ΔT_1_) correlated with the percentage of cells positive for the antigen Ki67 (a marker of cycling cells), but not with other markers such as apoptosis, necrosis or blood volume, all of which showed no consistent change with drug-treatment [7]. Recently, a preclinical study in a neuroblastoma mouse model treated with three different drugs showed a consistent decrease in T_1_[8], and a clinical study reported a small decrease in T_1_ in patients with colorectal cancer metastasis undergoing bevacizumab therapy [9].

To investigate further what ΔT_1_ reflects about the tumour biology, we have compared ΔT_1_ with magnetic resonance spectroscopy (MRS) markers of proliferation and apoptosis *in vivo*[10], as well as histology and immunohistochemistry *ex vivo* following treatment with the allosteric mTOR inhibitor, everolimus (Afinitor) in two different murine tumour models, RIF-1 and B16/BL6. Everolimus was selected for these studies because although the drug has significant clinical activity in several different types of cancer, there is currently no confirmed molecular marker that can stratify the patient population [11]. Using the RIF-1 model, we demonstrate that ΔT_1_ is a highly sensitive and specific predictor of response to everolimus and also the microtubule stabilizer patupilone. Collectively, these data further suggest that incorporation of T_1_ measurements in clinical trials should be an important aid to drug development and optimization of existing drugs.

## Methods

### Tumour Models

All animal experiments were carried-out strictly according to the local Swiss animal welfare regulations. The protocol was approved by the local veterinary authorities (Kantonales Veterinäramt Basel-Stadt, permit number 1974). C3H/He female mice (20–25 g) and C57/BL6 mice (20 g) were obtained from Charles River (France) and were acclimatized to local conditions for at least one week prior to experiments. Three experiments were performed in the RIF-1 fibrosarcoma model in C3H/He mice, one experiment was performed in the B16/BL6 melanoma model in C57/BL6 mice. All animal experiments were performed under isoflurane anesthesia, and every effort was made to minimize suffering.

Tumour volume (TVol) and animal body-weight (BW) measurements were made at least twice per week including just before treatment (baseline) and the endpoint. TVol was determined using calipers to measure three orthogonal dimensions and applying the formula: l*h*w*π/6.

#### Murine RIF-1 fibrosarcoma

Freshly cultured RIF-1 tumour cells were injected subcutaneously (5 × 10^6^ in 50 μL phosphate-buffered saline) in the upper thigh of anesthetized C3H/He mice, as previously described [12]. After 2 weeks, tumours were of sufficient size (at least 200 mm^3^) for the studies and were divided into two equal groups and treated daily with compound or vehicle. Experiment 1: treatment with everolimus (n = 7) compared to vehicle (n = 7), experiment 2: treatment with everolimus (n = 8) compared to vehicle (n = 8), experiment 3, previously published in [7]: three different doses of patupilone (each group n = 8) compared to vehicle (n = 8).

#### Murine B16/BL6 melanoma

Freshly cultured B16/BL6 tumour cells expressing the enzyme luciferase were injected intra-dermally (5 x 10^4^ in 1 μl) into the dorsal pinna of both ears of anesthetized C57/BL6 mice as previously described [12,13]. These black melanoma cells rapidly metastasize from the primary ear tumour to the regional lymph-nodes, in particular the neck. After 2 weeks, mice were divided into two equal groups (n = 10) and treated daily with everolimus (10 mg/kg p.o.) or vehicle for 6 days (experiment 4). MRI was performed on the metastasis in the cervical lymph nodes on day 5. In one mouse in the vehicle group there was no measurable lymph node metastasis.

### Compounds/drugs and their application

All compounds utilized in this study were obtained from the Novartis chemical department. The compounds and their respective vehicles were prepared each day just prior to administration to animals and the administration volume individually adjusted based upon animal body weight. Everolimus (RAD001) was obtained as a microemulsion and was freshly diluted in a vehicle of 5% glucose and administered by oral gavage (p.o.) to mice daily in a volume of 10 ml/kg at 10 mg/kg. Patupilone (epothilone B, EPO906) was dissolved in polyethylene glycol-300 (PEG-300) and then diluted with physiological saline (0.9% w/v NaCl) to obtain a mixture of 30% (v/v) PEG-300 and 70% (v/v) 0.9% saline. Treatment with vehicle (PEG-300/saline) or patupilone (3, 5 or 6 mg/kg) was once weekly using an i.v. bolus of 2–3 sec in the tail vein.

### Experimental design

Mice were divided into different treatment groups so that each group had the same mean TVol, and magnetic resonance (MR) measurements were made before treatment (baseline) i.e. day 0 and at the endpoint. For everolimus, the endpoint was day 5, and for patupilone it was day 7. T_1_ was measured in all four experiments. MRS was performed in experiment 1 only. Bioluminescence was measured *ex vivo* in experiment 4 (see below). At the end of everolimus-experiment 1, animals were sacrificed by CO_2_ inhalation, the tumours ablated and prepared for histology and immunohistochemistry (IHC) as previously described [7].

### Magnetic Resonance *in vivo*

Animals were anaesthetised using 1.5% isoflurane (Abbott, Cham, Switzerland) in a 1:1 mixture of O_2_/N_2_ and placed on an electrically warmed pad for canulation of one lateral tail-vein as previously described [7]. MRI experiments were performed on a Bruker DBX 47/30 or Avance 2 spectrometer (Bruker Biospin, Ettlingen, Germany) at 4.7 T equipped with a self-shielded 12 cm bore gradient system.

#### Quantitative T_1_ imaging

The spin–lattice relaxation of protons (T_1_) was measured with an inversion recovery (IR) TrueFISP (true fast imaging with steady state precession sequence, [14]) imaging sequence as previously described [7]. The basic sequence was a series of 16 TrueFISP images acquired at a time interval, TI, following a global 180° inversion pulse (TI = 210 ms to 5960 ms in 324 ms increments). Each TrueFISP image (one slice containing the central part of the tumour) was acquired with a flip angle α of 30°, a matrix size of 128 × 96, a field-of-view of 30 × 22.5 mm^2^, a slice thickness of 2 mm, an echo time (TE) of 1.7 ms, and a repetition time (TR) of 3.4 ms. Pixelwise T_1_ calculation was done using the method described in [15]. A region of interest (ROI) comprising the entire tumor was drawn manually on the resulting T_1_ map and the mean T_1_ of the central tumour slice was calculated in this ROI. MR image analysis was performed off-line with in-house written software based on IDL 6.0 programming environment (Research Systems Inc., Boulder, CO, USA).

#### ^1^H-MR spectroscopy

Localized shimming with FASTMAP method was performed on a 2.5 mm^3^ voxel to obtain line widths of <20 Hz. Point resolved spectroscopy (PRESS) experiments at the same voxel position (voxel size = 8 mm^3^, TE = 20 msec, TR = 1500 msec, SW = 4000 Hz, TD = 2048, with external volume suppression) were performed. One spectrum was acquired with water suppression (400 averages) and one spectrum without water suppression (1 average). The total time for MRS was 10–12 min for each mouse. The water signal (one peak) of the non-suppressed spectrum was used as an internal reference for relative quantification of metabolites using the metabolite to H_2_O ratio (Cho/H_2_O for choline, etc.). Peaks in the water-suppressed spectrum were identified by their chemical shifts, so total choline (Cho) was at 3.2 ppm, CH_3_-lipids at 0.9 ppm, CH_2_-lipids at 1.3 ppm, creatine at 3.0 ppm, and polyunsaturated fatty-acids (PUFA) at 5.3 ppm and very weakly at 2.8 ppm; however the latter peak was not used for any calculations.

### Histology and immunohistochemistry

A tumour slice of 3–4 mm thickness was cut from the largest circumference of the tumour, immersion-fixed in 4% (w/v) phosphate-buffered formalin (pH 7.4; J.T. Baker, Medite, Service AG, Dietikon, Switzerland) at 4°C for 24 hours and processed into paraffin as previously described [4]. IHC was performed on paraffin sections of 3 μm using the following antibodies for detection of (i) cleaved Caspase-3 (rabbit polyclonal antibody #9661, Cell Signaling, Danvers, MA, USA) (ii) Ki67 (rat monoclonal antibody, clone TEC3, #M7249, DAKO, Glostrup, Denmark) and (iii) CD31 (rabbit polyclonal antibody, #E11114, Spring Biosciences, Pleasanton, CA, USA).

### Image acquisition and analysis of histological slices

For quantification, the entire section was scanned using the MiraxScan system (Carl Zeiss AG, Jena, Germany). The absolute size of viable, necrotic and complete tissue areas was measured on the full scans using MiraxViewer software (Carl Zeiss AG, Jena, Germany). Quantification of Ki67 positive and negative nuclei in the complete viable areas was performed in a fully automated manner with TissueMap software at Definiens AG, Munich, Germany. Results were summarized as the total area, percentage-viable and percentage-necrotic area, total number of nuclei and the cell density (number of nuclei per mm^2^) in both the viable and total (including therefore necrotic) areas. Cleaved caspase-3 positive particles were quantified as positive pixels per total pixels in a semi-automated fashion with the AnalySIS® FIVE software (OlympusSIS, Münster, Germany) on six images (346.7 x 260 μm^2^ each) per section excluding necrotic areas and border zones of necrotic areas. CD31 stained slides were scanned with the Aperio ScanScopeXT slide scanner (Aperio, Vista, CA, USA) and vessels were quantified with the Aperio ImageScope software, using the Microvessel Analysis v1 Algorithm.

### Bioluminescence

Because of the black pigmentation of the C57/BL6 mice, bioluminescence (BioL) could not adequately be measured *in vivo* and therefore was determined *ex vivo* as follows. After 6 days, the cervical lymph-nodes were removed and weighed and then iced. Individual lymph-nodes were homogenized at 4˚C with 1 mL cold phosphate-buffered saline (without Ca^2+^ and Mg^2+^), rinsed in the same buffer, and 200 μl triplicates placed in a 96-well plate with 50 μl D-luciferin (1 mg/mL). BioL was measured at an emission wavelength of 560 nm using the imaging chamber of the IVIS™ system (Caliper Life Sciences Inc, Hopkinton, MA, USA) for 1 min at room temperature.

### Data analysis

Results are presented as mean ± SEM except where stated and all available data are shown. The T/C ratio is commonly used to quantify tumour growth inhibition, where T and C represent the means of the relative tumour volumes (tumour volume divided by its initial volume) of the treatment and control mice, respectively [16]. Longitudinal changes, such as in tumour volume (ΔTVol) or in T_1_ (ΔT_1_), were expressed as change between endpoint and baseline divided by value at baseline (fractional change in %). The T/C ratio was calculated for all parameters. For parameters measured at one time point only (such as histological read-outs), T/C was calculated as ratio of means of treatment and control mice, respectively. Differences between groups were analysed using a 2-tailed t-test. For the *in vivo* biomarker analyses which involved longitudinal analyses in the same animals, differences were analysed by a) 2-way repeated measures ANOVA and b) t-test at the endpoint; the latter method is therefore associated with the respective T/C. The different dose groups in experiment 3 were tested with 1-way ANOVA vs. control group. Quantification of the linear-relationship between the various parameters measured *in vivo* and *ex vivo* were analysed by Pearson’s correlation to provide the correlation coefficient (r) and the significance (p). Application of the non-parametric Spearman’s correlation did not affect the results except in one case (see Results). To facilitate comparison of PUFA levels which were not always detectable, a 2-sided Fisher’s exact test was also used. For all tests, the level of significance was set at p < 0.05 (two-tailed) where *p < 0.05, **p < 0.01, ***p < 0.001 versus vehicle.

To determine the sensitivity and specificity of a change in the imaging marker T_1_ as a marker of tumour response to treatment, receiver-operator curves (ROC) were generated [17,18] using Graphpad Prism (GraphPad Software, La Jolla, CA, USA) considering mice treated with the drugs everolimus (experiment 1 and 2) or patupilone (experiment 3). Briefly, responders (R) to drug-treatment were defined as showing no change or regression, in TVol, defined as ΔTVol ≤ 10%, while all others were considered non-responders (NR). Each of these tumours was then classified as R or NR by the ΔT_1_ at different discrete cut-offs to generate at each ΔT_1_ value a table of positive and negative predictions for determination of specificity and sensitivity at each value. The plot of 1-specificity versus sensitivity generated the ROC curve and the area under this curve (AUC) was quantified by the trapezoidal method.

## Results

### Effects of everolimus on MRI and MRS biomarkers in RIF-1 tumours *in vivo*

**Figure 1:**
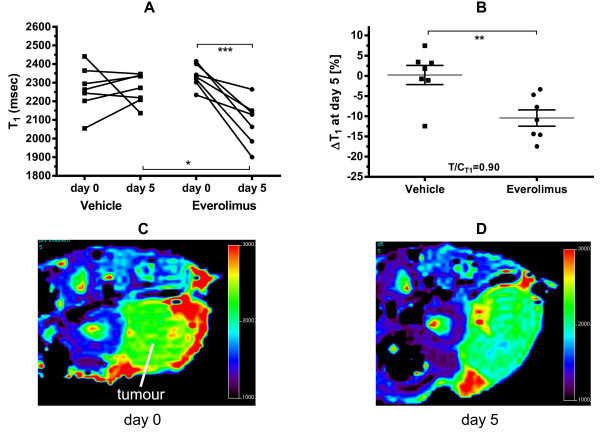
**Everolimus decreases the T**_**1 **_**of RIF-1 tumours.** C3H mice bearing RIF-1 tumours were treated with everolimus (10 mg/kg/day) or vehicle for 5 days and the spin–lattice relaxation of protons (T_1_) in tumours was measured on day 0 and at the endpoint day 5. Results show the individual values for each tumour, n = 7 per treatment group **(A)** and the mean ± SEM fractional change ΔT_1_ for each treatment **(B)**, where *p < 0.05, **p < 0.01, ***p < 0.001 as shown. Panel **C** &**D** show an MRI-derived T_1_ map from a representative RIF-1 tumour (arrow) before **(C)** and after **(D)** treatment with everolimus.

Murine RIF-1 tumours grew rapidly having a 2.5-fold increase in tumour volume after just 5 days, but daily treatment with everolimus (10 mg/kg p.o.) strongly inhibited tumour growth causing essentially stable disease at the 5 day endpoint with a T/C of 0.05 (Table 1). Both groups had a significant difference in TVol at baseline (slightly larger TVol in the treatment group), but this did not have an effect on the results, as can be seen in Table 1. Quantification of tumour T_1_ by MRI at baseline gave a mean ± SD of 2301 ± 100 msec (both groups, n = 14) which showed no significant change in vehicle-treated mice, but was reduced in 7/7 mice treated with everolimus providing a small but highly significant mean decrease of 10 ± 2% after 5 days treatment (Figure 1, Table 1).

**Table 1:** **Summary of RIF-1 measurements *in vivo *** **and *ex vivo***

**Parameter**	**Vehicle**		**Everolimus**		**T/C**
	**Day 0**	**Day 5**	**Day 0**	**Day 5**	
TVol (mm^3^)	447 ± 75	1118 ± 150	706 ± 85	736 ± 100	0.05***
T_1_ (msec)	2266 ± 46	2265 ± 30	2335 ± 23	2090 ± 45	0.89**
choline/H_2_O [a.u.]	2.72 ± 0.18	2.72 ± 0.2	2.6 ± 0.12	1.82 ± 0.32	0.73
CH_2_/H_2_O [a.u.]	6.44 ± 0.85	7.3 ± 1.17	6.89 ± 0.35	11.0 ± 1.5	1.3
CH_3_/H_2_O [a.u.]	2.56 ± 0.43	2.86 ± 1.74	3.06 ± 0.34	4.87 ± 0.72	1.2
PUFA/H_2_O [a.u.]	0.1 ± 0.1	0.37 ± 0.24	0.25 ± 0.25	0.8 ± 0.39	2.2
creatine/H_2_O [a.u.]	1.27 ± 0.15	1.14 ± 0.05	1.07 ± 0.06	0.89 ± 0.09	0.9
total area (mm^2^)	-	65.8 ± 10.0	-	43.6 ± 5.6	0.66
viable area (mm^2^)	-	52.1 ± 9.4	-	33.1 ± 4.7	0.64
%necrosis	-	21.5 ± 6.1	-	24.9 ± 3.3	1.16
total cells (thousand)	-	489 ± 82	-	328 ± 48	0.67
cells/viable mm^2^	-	9671 ± 454	-	9923 ± 562	1.03
%Csp^+^ area	-	0.39 ± 0.07	-	0.27 ± 0.02	0.7
BV per mm^2^	-	728 ± 159	-	768 ± 37	1.06
%Ki67^+^ cells	-	44.6 ± 2.5	-	26.5 ± 1.0	0.59***
Ki67^+^cells/mm^2^	-	4274 ± 205	-	2614 ± 141	0.61***
Ki67^-^ cells/mm^2^	-	5396 ± 457	-	7309 ± 462	1.35*

C3H mice bearing RIF-1 tumours were treated with everolimus (10 mg/kg/day, n = 7) or vehicle (n = 7) for 5 days prior to sacrifice. Tumour volume, T_1_ and the various MRS parameters were quantified prior to treatment (day 0) and after 5 days treatment. Animals were then culled and the tumours ablated and prepared for immunohistochemistry as described in Methods. After immunostaining for Ki67 and cleaved caspase-3, the entire tumour sections were scanned to provide viable and necrotic areas in mm^2^. The number of cells and the number and percentage staining for Ki67 and caspase-3 is shown only for the viable area but essentially identical results were obtained if the entire area was used. Blood vessels (BV) were quantified from CD31 stained slices. All results show the mean ± SEM for each tumour, significant changes are indicated by emboldened numbers where *p < 0.05, **p < 0.01, ***p < 0.001 comparing the two treatment group means (2-tailed t-test).

**Figure 2:**
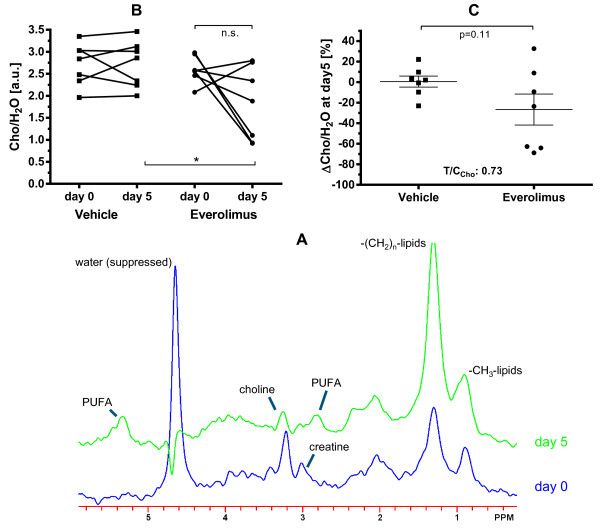
**Everolimus decreases the Cho/H**_**2**_**O ratio of RIF-1 tumours.** C3H mice bearing RIF-1 tumours were treated with everolimus (10 mg/kg/day) or vehicle for 5 days and the ratio of total choline to (unsuppressed) water (Cho/H_2_O) in tumours was measured on day 0 and at the endpoint day 5. Panel **A** shows a ^1^H-MRS spectrum from a representative RIF-1 tumour before and after treatment with everolimus. Graphs show the individual values for each tumour, n = 7 per treatment group **(B)** and the mean ± SEM fractional change ΔCho/H_2_O for each treatment **(C)**, where *p < 0.05 as shown.

^1^H-MRS on the same tumours at the same time-points was also performed to provide signals for total choline, creatine, as well as polyunsaturated lipids (PUFA) and saturated (CH_2_ and CH_3_) lipids (Figure 2A). The strong water signal (unsuppressed) was used to provide quantitative information as ratios (see Methods). This data showed that creatine and saturated lipids did not change in either treated-group (Table 1). However, total choline (Cho) in most cases (5/7) decreased in everolimus-treated tumours, with a mean change in choline ΔCho/H_2_O of 27 ± 15% (statistically significant only in ANOVA, not in t-test; Figure 2B,C). The PUFA peaks were broad and of low intensity (Figure 2A) and were only detectable at baseline in 1/7 tumours for each group. However, after 5 days treatment they were more prevalent, permitting quantification in 2/7 vehicle-treated and 5/7 everolimus-treated tumours which showed a T/C of 2.2 (Table 1). This data was obviously rather variable and scarce due to the limit-of-detection by MRS and there was no significant difference between the two groups although a two-way repeated-measures ANOVA (as used for T_1_ and Cho) showed that everolimus significantly increased PUFA (p = 0.007), a parameter that has also been associated with apoptosis [10].

### *Ex vivo* analyses of everolimus on RIF-1 tumours

**Figure 3:**
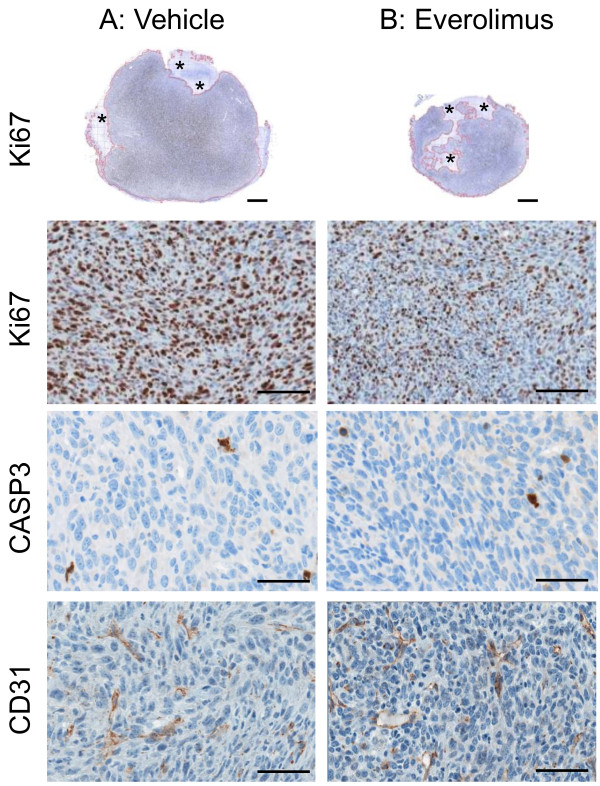
**Immunohistochemistry in RIF-1 tumours after everolimus or vehicle treatment.** C3H mice bearing RIF-1 tumours were treated with vehicle (**A**, left column) or everolimus at 10 mg/kg/day (**B**, right column) for 5 days prior to sacrifice (n = 7 per group). Tumours were ablated and prepared for immunohistochemistry as described in Methods. The entire sections were scanned. Ki67-stained slices of one representative tumour from each treatment (scalebar = 1 mm) are shown in row 1. Viable tumor tissue is outlined in red and necrosis regions are marked with asterisks. Percentage of Ki67-positive cells was 46% and 26% and the total tumour area was 43.9 mm^2^ (8% necrosis) and 22.4 mm^2^ (19% necrosis) for the vehicle-treated and everolimus-treated mice, respectively. Magnified sections stained for Ki67 (scalebar = 50 μm), cleaved caspase-3 (CASP3, scalebar = 25 μm), and CD31 (scalebar = 25 μm) are shown below in row 2, 3, and 4, respectively. There was no difference between the groups in apoptosis (CASP-3 staining) and blood-vessel density (CD31 staining).

At the endpoint, the ablated tumours were prepared for histology. Since everolimus inhibited tumour growth, there was of course a reduction in the total and viable area and consequently a reduction in the total cell number examined by IHC comparing everolimus-treated to vehicle-treated mice (Table 1, Figure 3), although these did not quite reach significance (p = 0.1). The cell density (cells per mm^2^) in the viable (or total) area and the %-necrosis was the same in each group (Table 1). The number of cells positive for the proliferation marker Ki67 was relatively high in vehicle-treated mice (45 ± 3%) and everolimus caused a clear and highly significant decrease in the total number and %Ki67^+^ cells to 27 ± 1% (Table 1; Figure 3, first and second row). Correspondingly, there was a significant increase in the number of Ki67^-^ cells. In contrast, there was a very low level of apoptosis as measured by caspase-3 staining in these tumours (<1%) and this was not affected by everolimus treatment (Table 1; Figure 3, third row). The number of blood-vessels (BV) per slice was rather variable, particularly in the vehicle-group, and a comparison of the blood-vessel density between treatment groups showed no effect from everolimus (Table 1; Figure 3, last row CD31).

### Relationships between RIF-1 biomarkers and tumour response

**Figure 4:**
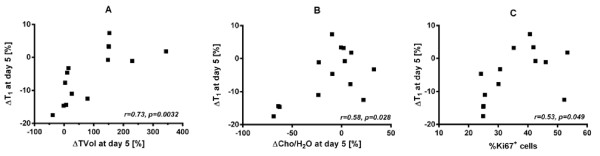
**Inter-relationships of biomarkers following everolimus or vehicle treatment of RIF-1 tumours.** Graphs **A-C** show Pearson correlations with the associated r and p values between the fractional change in T_1_ (ΔT_1_ ) and the fractional change in tumour volume (ΔTVol), the fractional change in total choline (ΔCho/H_2_O), and percentage of Ki67 positive cells, respectively, after 5 days of everolimus or vehicle treatment (n = 7 per group) of C3H mice bearing RIF-1 tumours.

As previously observed for several different experimental models and drugs, including everolimus [7], the change in T_1_ (ΔT_1_) was highly significantly (p = 0.0032) positively correlated with the change in RIF-1 tumour volume (ΔTVol), see Figure 4A, but there was no significant correlation between ΔCho/H_2_O and ΔTVol. Correlation of ΔT_1_ and ΔCho/H_2_O reached significance (r = 0.58, p = 0.028), see Figure 4B, although not when a Spearman correlation was applied (r = 0.43, p = 0.13). ΔT_1_ showed a significant positive correlation with the %Ki67^+^ cells (Figure 4C), a similar level of correlation existed between %Ki67^+^ and ΔCho/H_2_O (r = 0.56, p = 0.036); and of course negative correlation with the %Ki67^-^ cells (graphs not shown). There was no significant relationship between basal T_1_ value and ΔTVol in keeping with previous observations in many different preclinical models [7] that basal T_1_ cannot predict response. The final T_1_ or ΔT_1_ was unrelated to cell density, or indeed the total number of cells, with flat lines of correlation and wide scatter (results not shown). Thus, ΔT_1_ was unrelated at the endpoint to cell density or the extracellular space but was related to the remaining number of proliferating cells (and negatively correlated to the number of non-proliferating cells). PUFA levels, which also tended to change with treatment could not be formally correlated since the data was categorical, but a Fisher’s exact test showed a significant association between Cho and PUFA (p = 0.02) i.e. proliferation decreased as apoptosis increased.

### Sensitivity and specificity of ΔT_1_ as a response biomarker in RIF-1 tumours

**Figure 5:**
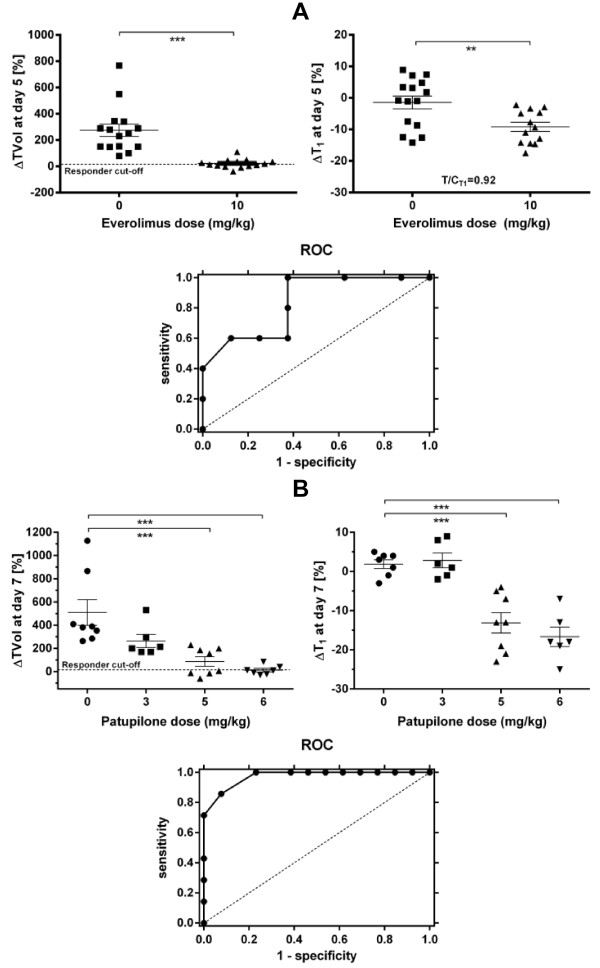
**Sensitivity and specificity of ΔT**_**1 **_**for everolimus and patupilone chemotherapy in the RIF-1 tumour model. A**. C3H mice bearing RIF-1 tumours were treated with everolimus (10 mg/kg/day) or vehicle for 5 days and TVol and the spin–lattice relaxation of protons (T_1_) in tumours was measured on day 0 and at the endpoint day 5 (n = 15 per group on day 0). In the everolimus group, one mouse died before endpoint and T_1_ measurement failed in another mouse on day 5. Results show the change in TVol (ΔTVol) (left panel) and the fractional change in T_1_ (ΔT_1_) (right panel) for each treatment on day 5, where **p < 0.01, ***p < 0.001 as shown. The ROC plots 1-specificity versus sensitivity for tumours treated with everolimus only and has an AUC = 0.84; the dashed line is the line of equivalence where the AUC = 0.5. **B**. C3H mice bearing RIF-1 tumours were treated with patupilone (3, 5 or 6 mg/kg i.v. bolus once) or vehicle and TVol and T_1_ in tumours was measured on day 0 and at the endpoint day 7 (n = 8 per group). In some mice, T_1_ could not always be determined and thus there were only n = 6 or 7 per group for analysis. Results show the ΔTVol (left panel) and the ΔT_1_ (right panel) for each treatment on day 7 where ***p < 0.001 as shown. The ROC plots 1-specificity versus sensitivity for tumours treated with different doses of patupilone and has an AUC = 0.97; the dashed line is the line of equivalence where the AUC = 0.5.

Correlations and linear regression provide indications of whether a biomarker could be used to predict response, but a receiver operating characteristic curve (ROC) can be more definitive in terms of the specificity and sensitivity of the marker. To generate such a curve for everolimus, data from experiments 1 and 2 was pooled from RIF-1 tumours (Figure 5A). The left panel shows the ΔTVol for mice treated with vehicle or everolimus and the right panel the ΔT_1_ in those tumours from day 0 to day 5 (n = 15 vehicle, n = 13 everolimus; in the everolimus group of experiment 2 one mouse died before endpoint and T_1_ measurement failed in another mouse). Responders to everolimus treatment were defined as showing a maximum change in ΔTVol of +10% (stable disease or regression), so giving 5R and 8NR and providing the ‘gold-standard’. Each of these 13 tumours was then classified as a R or NR by the ΔT_1_ using different discrete cut-offs ranging from −16.5% to −2.5% to generate at each ΔT_1_ value a table of positive and negative predictions for determination of specificity and sensitivity at each value, see for example Table 2A. The plot of 1-specificity versus sensitivity generated the ROC curve and the area under this curve (AUC) was quantified giving a value of 0.84 which is considered to be very good predictive ability [17].

**Table 2:** **T**_**1 **_**sensitivity and specificity tables for changes in RIF-1 tumour volume following everolimus or patupilone treatment**

**A.** Everolimus: using a ΔT_1_ of −8%			
	**ΔTVol**		
**ΔT**_**1**_	**Positive**	**Negative**	**Total**
**Positive**	**5**	**3**	**8**
**Negative**	**0**	**5**	**5**
**Total**	**5**	**8**	**13**
**B.** Patupilone: using a ΔT_1_ of −8%.			
	**ΔTVol**		
**ΔT**_**1**_	**Positive**	**Negative**	**Total**
**Positive**	**7**	**3**	**10**
**Negative**	**0**	**10**	**10**
**Total**	**7**	**13**	**20**

**A.** Everolimus: The prevalence (total positive/all) = 0.38. The sensitivity =1.0 and the 1- specificity = 0.38, giving a positive prediction value of 0.63 and a negative prediction value of 1.0 at this ΔT_1_ cut-off of −8%.

**B.** Patupilone: The prevalence (total positive/all) = 0.35. The sensitivity =1.0 and the 1- specificity = 0.23, giving a positive prediction value of 0.70 and a negative prediction value of 1.0 at this ΔT_1_ cut-off of −8%.

The same approach was used to analyse data already published [7] from the cytotoxic patupilone on the same RIF-1 tumour model. In this case, a dose–response was used (Figure 5B, left panel) and R and NR identified in the same way giving 7R and 13NR (from the 20 tumours). The right panel (Figure 5B) shows the ΔT_1_ in those tumours from day 0 to day 7 (n = 6-8 per dose). Using once more discrete ΔT_1_ values from −24% to 8.5% a table of positive and negative predictions was generated (see for example Table 2B) and the ROC plotted (Figure 5B). The AUC of this plot was 0.97 confirming outstanding predictive ability for ΔT_1_ in the RIF-1 model.

### Relationship between bioluminescence and T_1_ in B16/BL6 tumours

**Figure 6:**
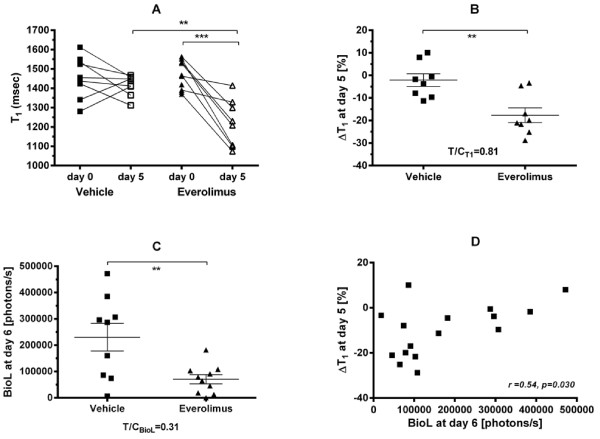
**Everolimus decreases the T**_**1 **_**and bioluminescence of cervical B16/BL6 melanoma metastases.** C57/BL6 mice bearing B16/BL6 melanomas were treated with everolimus (10 mg/kg/day) or vehicle for 6 days and the spin–lattice relaxation of protons (T_1_) in tumours was measured on day 0 and at the endpoint day 6. Lymph-nodes were removed and extracted for measurement of bioluminescence (BioL) as described in Methods. Results show the mean ± SEM, and the individual T_1_ values **(A)** and bioluminescence **(C)**, the fractional change in T_1_ (ΔT_1_) for the treatments **(B)**, and the Pearson correlations (with the associated r and p values) between BioL and ΔT_1_**(D)**, where **p < 0.01, ***p < 0.001 as shown.

Everolimus inhibited growth of B16/BL6 lymph-node metastases after 6 days daily treatment leading to a T/C ratio for the weight of the dissected metastases of T/C_weight_ = 0.60 and this was associated with a highly significant decrease in the T_1_ of the metastases of 19 ± 3% (Figure 6A,B). T_1_ measurement failed in one mouse treated with vehicle and in two mice treated with everolimus resulting in n = 8 ΔT_1_ values in each group. Bioluminescence from these lymph-nodes measured *ex vivo* was also significantly decreased (Figure 6C) and this correlated significantly with the ΔT_1_ (Figure 6D). Since the enzyme luciferase is only located within the melanoma cells, the bioluminescence should only reflect the viable tumour cells which supports the notion that a change in T_1_ is an indirect measurement of the viable and/or proliferating cell fraction.

## Discussion

We have previously shown that a small but highly significant decrease in the mean spin–lattice relaxation of protons (T_1_) of experimental tumours induced by various different types of chemotherapy is strongly correlated with the change in tumour volume and also the immunohistochemical proliferation marker Ki67 [7]. Furthermore, in the RIF-1 model the antimetabolite 5FU also decreased levels of the proliferation marker choline and this too was correlated with the change in T_1_ (ΔT_1_). The data presented here on RIF-1 and B16/BL6 tumours confirm these observations for the allosteric mTOR inhibitor everolimus, providing further evidence that ΔT_1_ reflects the number of remaining proliferating tumour cells following successful chemotherapy. The greater the decrease in T_1_, the lower the percentage of proliferating cells after therapy. In the previous report, a sample area (10%) of an *ex vivo* tumour slice was examined histologically, and thus, true cell density and also therefore the overall extracellular space could not be assessed either. In two models, using the cytotoxic patupilone on murine RIF-1 and rat mammary BN472, there were trends for cell-density to decrease by approx. 10% but neither reached significance [7]. In this report, we have made a detailed histological study of the effect of everolimus on RIF-1 tumours grown s.c. in murine C3H mice.

RIF-1 cells are sensitive to everolimus with an IC50 *in vitro* of 2.6 ± 1.6 nM (insensitive cells have an IC50 > 1 μM, see references [19,20]), but this is still not as sensitive as the endothelial cells which have IC50 <1 nM, which likely explains the fact that everolimus has anti-tumour cell as well as anti-angiogenic properties [19]. Daily treatment of mice bearing RIF-1 tumours caused tumour stasis, and consistent with this, histology at the endpoint of 5 days showed a decrease in total tumour area and a proportional decrease in the viable area of approx. 35% compared to vehicle (both not significant, p < 0.1). However, the total number of cells showed a similar trend for a decrease in proportion (p < 0.1) and thus the overall cell density in the viable areas was unchanged. Since necrosis was also not affected by everolimus (non-significant increase of 20%), this analysis showed that cell density and the extracellular space were unaffected. Many previous experiments *in vitro* and using human tumour xenografts *in vivo* have shown that T_1_ is sensitive to a) the amount of water in the extracellular space (but not intracellular) and b) the amount of protein in the water [21-25]. It is well recognised that inhibition of mTOR (the molecular target of everolimus) causes a decrease in cell size [26], because cell cycle progression is blocked at G1 thus inhibiting protein synthesis and cell growth. Consequently, cell density might not change, but the extracellular space could increase. Unfortunately we could not measure the average cell size because defining where one cell ends and another begins is difficult and there was no automatic programme for such an approach. But in any case, an increase in extracellular space would lead to an increase rather than a decrease in T_1_[21-25], suggesting that if cell size changes occurred they could not explain the T_1_ decrease that we have always observed following successful chemotherapy with many different agents [7]. This suggests to us, that tumour cell and vascular destruction leads to the release of proteins and also paramagnetic ions into the extracellular space which causes the decrease in T_1_; an effect which has been shown *in vitro*[22]. However, everolimus did not cause a decrease in the blood vessel density, as has been seen in several other tumour models [20,27-29], although this does not rule out an effect on the functional vasculature (previously measured as low in RIF-1 tumours [30]) and/or that early vascular changes had normalised by day 5. Also in this model, there was no clear evidence of increased tumour cell kill since caspase-3 levels were unaffected, although there did appear to be a strong trend for an increase in the PUFAs of everolimus-treated tumours which has been associated with apoptosis in other experimental models [10].

Immunohistochemistry (IHC) showed that approx. half of vehicle-treated RIF-1 tumour cells were positive for the nuclear antigen Ki67. Ki67 is considered to be a proliferation marker since it is expressed in all cycling cells (G1, S and G2M) but not therefore in cells in G0, and is a convenient IHC tool in the clinic for assessing tumour growth and response [31,32]. Given that the effect of mTOR inhibition is to block G1, it was not surprising that everolimus caused a marked decrease in the %Ki67^+^ cells whether expressed as total number or density and there was a proportional increase in the cells negative for Ki67. Everolimus also decreased levels of total choline (Cho) in RIF-1 tumours, which is another marker of viable and proliferating cells, in this case reflecting membrane turnover. Cho tends to be higher in tumour than normal tissue [33] and successful chemotherapy has also been shown to decrease in Cho in both experimental models [7,8,10] and the clinic [34,35]. In the RIF-1 tumours, these proliferation markers correlated significantly with each other as well as with the ΔT_1_, supporting the notion that ΔT_1_ is a surrogate of the remaining number of proliferating cells in a tumour after therapy even though our histological analysis suggests that it cannot be measuring cell number or density directly. Support for this hypothesis came from the B16/BL6 model treated with everolimus where again the decrease in bioluminescence, which measures viable tumour cell number, was correlated to the ΔT_1_.

It is worth repeating that we have found that six different types of chemotherapy including anti-metabolites, inhibitors of mTOR, microtubules, VEGF-R, PI3K and HSP90 [7] [and unpublished] in several different tumour-types implanted in both mouse and rat hosts, all showed a decrease in T_1_ in response to successful chemotherapy i.e. characterized by a significant change in TVol in comparison to vehicle-treated tumours. Furthermore, where a tumour was resistant to that particular type of chemotherapy (paclitaxel and patupilone), there was no change in T_1_[7]. This suggests that ΔT_1_ is a generic marker of tumour response, because, as discussed above, it reflects overall tumour destruction. But, what is the level of sensitivity and specificity i.e. how useful could such a method be in the clinic? To answer this question, we used receiver-operating-characteristic curves (ROC) to analyse two different models in which mice bearing RIF-1 tumours were treated with either a single dose of everolimus or three different doses of patupilone. In both cases, the ROCs had a large area-under-the-curve (AUC) of 0.84 and 0.97 which is considered of very good to outstanding predictive ability [17]. Consider for comparison, IHC markers of the mTOR pathway to predict everolimus activity *in vitro* using cell lines in which ROC-AUCs of 0.86-0.88 were determined [20]; also excellent predictive activity but no better or even lower than that we have shown here. Indeed, with our data, if a cut-off of an 8% decrease were selected (i.e. ΔT_1_ = −8%), then the sensitivity to both drugs would be perfect in this model at 1.0 i.e. providing a negative predictive value of 100%. In other words, after two MRI-scans one could completely eliminate from the study any tumours with a T_1_ decrease smaller than 8% since these should not benefit from further treatment.

## Conclusion

T_1_ showed a decrease in response to successful chemotherapy in several tumour models using various drugs. Analysis of histological and bioluminescence data from tumours treated with everolimus indicate that ΔT_1_ is a generic marker of tumour response reflecting overall tumour destruction and the decreased number of proliferating cells.

The excellent negative predictive value of ΔT_1_ suggests that the method should be tested in the clinic, for example wherever MRI is being used anyway to determine tumour size. Potentially, it provides the opportunity to stratify a patient population after the first cycle of treatment to increase the effective response-rate as well as to save resources by avoiding treatment of patients who are unlikely to respond.

## Abbreviations

AUC: Area under curve; BioL: Bioluminescence; BV: Blood vessel; BW: Body weight; Cho: Total choline; ΔCho/H2O: Change in ratio total choline to unsuppressed water; IR True FISP: Inversion recovery true fast imaging with steady state precession sequence; MoA: Mechanism of action; MRI: Magnetic resonance imaging; MRS: Magnetic resonance spectroscopy; IHC: Immunohistochemistry; PRESS: Point resolved spectroscopy; PUFA: Polyunsaturated fatty-acids; ROC: Receiver operating characteristic curve; ROI: Region of interest; T1: Spin–lattice MR relaxation time; ΔT1: Change in the spin–lattice relaxation time of water protons; ΔTVol: Change in tumour volume; T/C: Treated-value divided by control-value.

## Competing interests

All the authors are or were (CW) employees of Novartis Pharma AG, Basel, Switzerland. They declare no competing interests.

## Authors’ contributions

CW carried out the MRI and MRS studies, performed data and statistical analysis, and drafted the manuscript. PA carried out the MRI studies and critically revised the manuscript. MS performed and analysed histological and IHC studies. VR performed IHC studies and prepared figures with IHC data. SF took care of the animal model, the animal treatment, and carried out bioluminescence assessments. PM took care of the study design and coordination, performed data and statistical analysis, and drafted the manuscript. All authors read and approved the final manuscript.

## Pre-publication history

The pre-publication history for this paper can be accessed here:

http://www.biomedcentral.com/1471-2407/14/88/prepub
